# Assessing the potential and safety of *Myrtus communis* flower essential oils as efficient natural preservatives against *Listeria monocytogenes* growth in minced beef under refrigeration

**DOI:** 10.1002/fsn3.1497

**Published:** 2020-03-11

**Authors:** Wissal Dhifi, Sabrine Jazi, Marc El Beyrouthy, Carmen Sadaka, Wissem Mnif

**Affiliations:** ^1^ LR17‐ES03 Physiopathology, Food and Biomolecules Higher Institute of Biotechnology of Sidi Thabet Biotechpole Sidi Thabet Ariana Tunisia; ^2^ Faculty of Sciences of Bizerte University of Carthage Carthage Tunisia; ^3^ Department of Agriculture and Food Engineering School of Engineering Holy Spirit University of Kaslik Jounieh Lebanon; ^4^ Faculty of Medicine American University of Beirut Beirut Lebanon; ^5^ Department of Chemistry Faculty of Sciences and Arts in Balgarn University of Bisha Bisha Saudi Arabia; ^6^ ISBST, BVBGR‐LR11ES31 Biotechpole Sidi Thabet University of Manouba Ariana Tunisia

**Keywords:** biological activities, food poisoning, *Listeria monocytogenes*, listeriosis, *Myrtus communis* L., nisin, preservative, synergism

## Abstract

In this research, the chemical composition and biological properties of Tunisian *Myrtus communis* (*Mc*EO) flowers were investigated. The antibacterial effect of *Mc*EO toward some bacteria was assessed, alone and in combination with nisin. The major components of *Mc*EO were α‐pinene, 1,8‐cineol, limonene, and linalool. *Mc*EO exhibited cytotoxicity toward HepG2 and MCF‐7 cell lines. The microbiological data showed that Gram‐positive bacteria were more susceptible to *Mc*EO. *Mc*EO had a bactericidal effect against *L. monocytogenes*. *Mc*EO is able to prevent lipid oxidation, microbial development at noncytotoxic concentrations, when used alone or in combination with nisin. It can improve sensory attributes within acceptable limits and improve the conservation of shelf life of minced beef meat during the 4°C storage period. The most potent preservative effect was obtained with the mixture: 0.8% *Mc*EO with 500 IU/g of nisin. This combination may be a good alternative for the development of natural preservatives.

## Graphical abstract

This research investigates the capacity of essential oils extracted from the Tunisian Myrtus communis (McEO) flowers as a natural alternative to preserve minced beef.

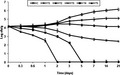

## 1 INTRODUCTION

Foodborne illnesses or food poisoning is a growing public health problem worldwide (de Jesus, Frazão, Blank, & De Aquino Santana, 2016). Its common causes include the ingestion of contaminated food. The contamination may occur during different stages of the food production and handling processes including preharvest, postharvest, and conservation process. Foodborne listeriosis caused by *Listeria monocytogenes* is recognized among the most threatening and severe food poisonings that emerged during the last two decades (Alam et al., 2016; Castro et al., 2017). Unlike other common pathogens causing foodborne diseases, *L. monocytogenes* can grow at low temperatures, making food stored under refrigeration a high risk for listeriosis. Food such as soft cheese, cold‐smoked fish, and meat (especially deli meat, sausages, cooked, cured, and/or fermented meat products) are considered high‐risk food causing listeriosis. The use of chemical additives is therefore essential to prolong the shelf life and to prevent the growth by *L. monocytogenes* in refrigerated meat products (Mir, Masoodi, & Raja, 2017). Given the growing safety concerns associated with the use of synthetic additives and preservatives, natural products such as plant essential oils (EOs) and bacteriocins (e.g., nisin) gained popularity as an alternative for food preservation including that of meat (Alizadeh Behbahani, Tabatabaei Yazdi, Shahidi, Mortazavi, & Mohebbi, 2017; Ben Hsouna, Ben Halima, Smaoui, & Hamdi, 2017; Smaoui et al., 2016). Due to their antioxidant and antibacterial activities, bacteriocins and some EOs can act as natural preservatives (Smaoui et al., 2016).

Nisin is a bacteriocin secreted by *Lactococcus lactis* or *Streptococcus uberis* strains and is generally considered a safe added ingredient (Castro et al., 2017). While most bacteriocin peptides exhibit a narrow spectrum of activity, nisin is commonly used as meat preservative thanks to its large activity field against Gram‐positive bacteria including *L. monocytogenes* (Alizadeh Behbahani et al., 2017; Hansen & Sandine, 1994).

*Myrtus communis* L. commonly known as myrtle belongs to the Myrtaceae family. Myrtle has been traditionally used in folk medicine for its anantiseptic, disinfectant, anti‐inflammatory, and hypoglycemic properties (Jabri et al., 2016). Various parts of myrtle have been used in the preparation of cosmetics and as additives to alleviate the flavor of some food (Aleksic & Knezevic, 2014). Most studies available on myrtle focus only on the activity of its leaves and berries EOs (Bajalan & Pirbalouti, 2014), and not on its flower EOs. The antimicrobial and natural preservative potential of EOs have been investigated alone (Bellili et al., 2018; Ozcan, Sagdic, & Ozcan, 2003) and in combination with nisin (Gao et al., 2014; Solomakos, Govaris, Koidis, & Botsoglou, 2008). Nonetheless, the effect of EOS extracted from *Myrtus communis* (*Mc*EO) flowers in combination with nisin has not been investigated yet. The current study aims to (a) ascertain the chemical composition, antioxidant, and cytotoxic activities of *Mc*EO of Tunisian myrtle; (b) evaluate the antimicrobial activity of *Mc*EO against a panel of Gram‐positive and Gram‐negative bacteria; (c) evaluate the antimicrobial activity of *Mc*EO, alone and in combination with nisin, against *L. monocytogenes* in refrigerated raw minced beef meat; and (d) assess the effect nisin–*Mc*EO combination on the shelf life, microbiological/physicochemical properties, and sensory modifications of refrigerated raw minced beef meat.

## 2 MATERIALS AND METHODS

### 2.1 Plant material

Myrtle flowers were gathered during the month of June 2016 from the region of Elkef in Tunisia (located at 35.23°N, and 11.11°E). The flower identification was conducted by Professor Ferjani Ben Abduallah, botanist in the Faculty of Sciences of Sfax‐Tunisia.

### 2.2 Essential oil extraction

Hydrodistillation in a Clevenger for 3 hr of 1 kg of air‐dried myrtle flowers allowed the extraction of its essential oils. Dichloromethane (3 × 50 ml) and anhydrous sodium sulfate were used to extract and then dry the aqueous phase, respectively. Following filtration, a rotary evaporator was employed to eliminate the solvent by distillation under reduced pressure. The extracted oil was then refrigerated (4°C) in the dark (Ben Hsouna & Hamdi, 2012). The EO yields were determined based on the dry weight of plant material used as follows:

$$Mc\text{EO}\;\left(\%\;\;\text{v/w}\right)=\frac{\text{observed}\;\text{volume}\;\text{of}\;\text{oil}\;\left(\text{ml}\right)}{\text{weight}\;\text{of}\;\text{sample}\;(g)}\times 100.$$

### 2.3 Nisin preparation

The nisin stock solutions were made to a final concentration of 25,000 IU (International Units)/ml from 20 mg of purenisin (Nisaplin; 50 × 10^6^ IU/g; Danisco) dissolved in 0.02 N hydrogen chloride (HCl; Sigma‐Aldrich), filtered (using sterile 0.2 µm pore filters, Pall Life Sciences), and kept at −20°C. Frozen stocks of nisin solutions were later thawed at 25°C prior to experimentation, and dilutions to 500 IU/ml were made with sterile distilled water (Shahbazi, Shavisi, & Mohebi, 2016; Zhao et al., 2016).

### 2.4 Gas chromatography–mass spectrometry (GC‐MS)

The chemical composition of *Mc*EO was analyzed by GC‐MS (Agilent 6890N; Agilent Technologies), equipped with a capillary HP‐5MS column (60 m length, 0.25 mm diameter, 0.25 mm film thickness), and coupled with a mass selective detector (Agilent MSD5973 model; Agilent Technologies). The ionization voltage was 70 eV. The carrier gas was Helium (1.2 ml/min flow rate). The oven temperature was programmed for 1 min at 100°C, increased from 100 to 280°C at a rate of 5°C/min, and then set at 280°C for 25 min. The temperatures of the injector and detector were 250 and 310°C, respectively. The injection (µl) was conducted manually in the split mode (1:50 split ratio).

The identification of *Mc*EO components was done by comparing their mass spectra with those from two libraries: the Wiley Registry of Mass Spectral Data 7th edition (Agilent Technologies) and the library of the national institute of standards and technology 05 MS (NIST).

### 2.5 Cell culture conditions

The human liver (HepG2) and breast (MCF‐7) cancer cell lines were used in the cytotoxicity screens. These cell lines were grown in Roswell Park Memorial Institute (RPMI) 1640 medium (Thermo Fisher Scientific) supplemented with 10% (v/v) fetal calf serum (FCS) and 2 mM L‐Glutamine in tissue culture flasks (Nunc, Thermo Fisher Scientific) and passaged twice a week. Cell lines were preserved at 37°C ± 5% CO_2_.

### 2.6 MTT test

The proliferation rates of HepG2 and MCF‐7 cell lines following exposure to *Mc*EO were established by the 3‐(4,5‐dimethylthiazol‐2‐yl)‐2,5‐diphenyl tetrazolium bromide (MTT) assay (Hsouna et al., 2011). HepG2 (5 × 10^4^/ml) and MCF‐7 (5 × 10^4^/ml) cells were incubated in 96‐well plates (200 µl of cell suspension/well, Sigma‐Aldrich) for 72 hr in the presence and absence of *Mc*EO with serial dilutions (20–1,000 µg/ml). After 10 µl of MTT solution (5 mg/ml in PBS) (Sigma‐Aldrich) was added to each well, plates were incubated for 4 hr at 37°C in a CO_2_‐incubator (model 3154; Forma Scientific, Inc.). About 180 µl of medium was removed from every well, replaced with 180 µl of a 50:50 methanol/dimethyl sulfoxide (DMSO) solution, and mixed thoroughly on a plate shaker until the complete dissolution of crystals. The absorbance was then read at 570 nm using a microplate reader (ELX 800, Biotek). The assay was run in triplicate. The cell growth percentages were determined as follows:

$$\text{cell}\;\text{growth}\;\text{(\textbackslash{}\% )}=\frac{A_{\text{sample}}}{A_{\text{control}}}\times 100,$$

where *A* corresponds to the absorbance at 570 nm. The cytotoxicity was expressed as the concentration of *Mc*EO inhibiting 50% of cell growth (IC_50_).

### 2.7 Antioxidant testing assays

#### 2.7.1 2,2′‐diphenyl‐1‐picrylhydrazyl (DPPH) radical scavenging activity

Radical scavenging potential of *Mc*EO under study was determined using DPPH free radical scavenging assay, as previously described by Ben Hsouna et al. (2017) and Bellili et al. (2018). Ascorbic acid served as positive control. The different concentrations of *Mc*EO (1, 10, and 100 µg/ml) and ascorbic acid were prepared in methanol. About 0.25 ml of DPPH radical solution (0.2 mM) was added to the reaction mixture. The percentage of free radicals inhibition in percentages was calculated as follows:

$$\text{DPPH}\;\text{radical}\;\text{scavenging}\;\text{activity}\;\text{(\textbackslash{}\% )}=1-\frac{A_{\text{sample}}}{A_{\text{negative}\;\text{control}}}\times 100,$$

where *A* corresponds to the absorbance at 517 nm.

#### 2.7.2 β‐Carotene bleaching assay

The β‐carotene bleaching method defined by Solomakos et al. (2008) was used to determine the antioxidant activity of *Mc*EO. Tests were run in triplicate.

### 2.8 Antimicrobial activity

#### 2.8.1 Microorganisms, growth conditions, and test method

The antimicrobial activity of *Mc*EO was assessed by the agar well diffusion and the broth microdilution methods against a panel of reference pathogenic bacteria consisting of six different Gram‐positive strains (including *Bacillus cereus* ATCC14579, *B. subtilis* ATCC6633, *Enterococcus faecalis* ATCC 29212, *L. monocytogenes* ATCC 19117*, Staphylococcus aureus* ATCC 25923, and *S. epidermidis* ATCC 12228) and three Gram‐negative bacteria (*Escherichia coli* ATCC 25922, *Pseudomonas aeruginosa* ATCC 9027, and *Salmonella enterica* ATCC 43972). The strains were grown for 12–14 hr at 37°C in sterile Mueller–Hinton broth (BioRad). The inoculums were prepared by dilution of that culture to ~10^7^ colony‐forming units (CFU)/ml in a sterile saline solution (Ben Hsouna & Hamdi, 2012). All the tests detailed in the below sections (“Agar well diffusion method” and “Minimum inhibitory concentration (MIC) and minimum bactericidal concentration (MBC)”) were run in triplicates.

##### Agar well diffusion method

About 100 μl of inoculum was evenly spread on the surface of Mueller–Hinton agar plates. Sterile Pasteur pipette was used to punch 6‐mm wells into the agar plates after they had aseptically dried. *Mc*EO was dissolved in a 1:9 DMSO/water solution and diluted to a final concentration of 50 mg/ml with sterile water. About 50 μl of the prepared *Mc*EO solution was placed into the punched wells. Plates were then incubated at 37°C for 24 hr. About 50 μl of each of gentamicin (10 µg) and 1:9 DMSO/water (1:9) solution (50 μl) served as positive and negative controls, respectively. The test zone diameter of growth inhibition around the punched well was measured.

##### Minimum inhibitory concentration (MIC) and minimum bactericidal concentration (MBC)

The broth microdilution method was employed to determine the MICs of *Mc*EO (Sadaka, Kanellos, Guardabassi, Boucher, & Watts, 2016; Sfeir, Lefrançois, Baudoux, Derbré, & Licznar, 2013). The essential oil concentration ranges were 400, 300, 200, 100, 90, 80, 70, 60, and 50 µl/ml. Negative controls consisted of a DMSO and water solution (at a 1:9 DMSO‐to‐water ratio). About 10 μl of bacterial inoculum from the dilution corresponding to the MIC was therefore plated onto sheep blood agar (Thermo Fisher scientific) to determine viable CFU/ml (Sfeir et al., 2013). Incubation was done at 37°C for 24 hr. The MBC/MIC ratio was calculated to assess the type of antimicrobial effect of *Mc*EO (Soro, Kone, & Kamanzi, 2010).

### 2.9 Sample preparation

Raw minced beef meat was purchased from a supermarket in Sfax (Tunisia) and transported to the laboratory under refrigeration conditions within less than 30 min. Each meat sample was divided into six as follows: a control sample (C, untreated samples), two samples (T1 and T2) treated with *Mc*EO (at 0.4 and 0.8%, respectively), a sample (T3) treated with nisin (at 500 IU/g), and two samples (T4 and T5) treated with a combination of *Mc*EO and nisin (at 0.4%: 500 AU/g and 0.8%: 500 AU/g *Mc*EO to nisin concentrations, respectively). The *Mc*EO was dissolved in 10% DMSO, filtered (0.22 µm black polycarbonate Millipore filters; Merck KGaA), added to concentrations of 1 MIC and 2 MIC corresponding to 0.4% and 0.8% v/w of meat, respectively, and mixed to distribute the microorganisms equally (Ben Hsouna et al., 2017; Smaoui et al., 2016). Each sample formed therefore a homogeneous mixture that was stored under vacuum conditions in plastic bags to make up three replicates. Samples were then refrigerated for 21 days at 4°C.

### 2.10 Physicochemical analysis

#### 2.10.1 pH determination

Five grams of each sample were homogenized in 50 ml of distilled water (pH 7.00), filtered, and then subjected to pH measures (Ben Hsouna et al., 2017).

#### 2.10.2 Thiobarbituric acid‐reactive substances value (TBARS)

The distillation method was employed to measure TBARS and evaluate the oxidation of lipids in minced beef meat samples (Eymard et al., 2005). Results were given in mg of malonaldehyde (MDA) equivalents per kg of the sample (mg/kg) using the molar extinction coefficient of the MDA‐2‐thiobarbituric acid (TBA) at 532 nm (1.56 × 10^5^ M^−1^ cm^−1^).

### 2.11 Microbiological analysis

About 25 g of each meat sample was dissolved in 225 ml of sterile peptone water (0.1 g/100 ml) and homogenized in a stomacher for 90 s at room temperature. A serial 10‐fold dilution series was prepared in peptone water (0.1 g/100 ml). 100 µl of appropriate dilution of each sample was transferred on the agar plate to examine its microbiological quality using aerobic plate count (APC; plate count agar [PCA] plates incubated at 30°C for 48 hr), psychrotrophic bacterial count (PTC; PCA plates incubated at 7°C for 10 days), and *Enterobacteriaceae* counts by the pour plating method (Violet Red Bile Glucose agar plates incubated at 37°C for 48 hr; Smaoui et al., 2016).

To estimate the effect of *Mc*EO tested individually and in combination with nisin toward *L. monocytogenes* ATCC 19117 during 21 days at 4°C, minced beef meat samples were each inoculated with 100 ml of cell suspension of *L. monocytogenes*, containing 10^6^ CFU/ml.

The stored samples were examined following 1, 3, 6, 9, 12, 15, 18, and 21 days. The sample inoculated with the strain of interest, and to which sterile water was added served as negative control and was subject to the same storage conditions. *L. monocytogenes* were enumerated on PLACAM agar (Oxoid), and the colonies were counted after 24 hr of incubation at 30°C (Ben Hsouna et al., 2017). CFUs were counted in plates showing 30–300 colonies.

### 2.12 Sensory evaluation

Eighteen experienced panelists were chosen from the staff members of the University of Sfax to assess the color, appearance, odor, and overall acceptability of the minced meat samples. The assessment was performed using a nine‐point scale, where nine correspond to “like extremely” and one corresponds to “dislike extremely.” Values of or above five (which corresponds to “neither like nor dislike”) were regarded to as acceptable (Smaoui et al., 2016).

### 2.13 Statistical analysis

The SPSS 19 statistical software (SPSS Ltd.) was exploited to evaluate significant differences between the treated meat samples using the one‐way analysis of variance (ANOVA) and Turkey's post hoc test. Bacterial counts data were transformed into logarithms of the CFU per g of ground beef. Corresponding means, standard errors, and variances were analyzed. Differences between the mean values of the different treatments were assessed by the least significant difference test. A probability level of *p* < .05 was adopted to test the statistical significance of all the experimental data.

## 3 RESULTS AND DISCUSSION

### 3.1 Chemical composition of the essential oil

Identified *Mc*EO components (*n* = 17), their percentages, retention times (Rt), and their respective Kovats Indices are summarized in Table 1. The analysis of *Mc*EO composition identified 17 compounds accounting for 93.82% of the total oil. *Mc*EO yield was of 2.8% (v/w). The identified components were divided into three classes: hydrocarbon monoterpenes, oxygenated monoterpenes, and sesquiterpenes (Table 1). The predominant class in the *Mc*EO was that of hydrocarbon monoterpenes (59.25%) mainly represented by α‐Pinene (35.20%), 1,8‐Cineole (17%), and limonene (8.94%). Other major components identified in *Mc*EO included methyl eugenol (6.98%), linalool (6.17%), geranyl acetate (4.42%), terpenyl acetate (4.30%), transcaryophyllene (4.04%), α‐terpineol (3.86%), caryophyllene oxide (2.49%), myrtenyl acetate (1.26%), and myrcene (1.21%; Table 1). α‐Pinene was reported as the main constituent of *Mc*EO extracted from the flowers of Tunisian myrtle in this study (35.20%), and as the main constituent of *Mc*EO extracted from Italian myrtle cones and leaves (30% and 28.5%, respectively), it was reported as a minor constituent of *Mc*EO extracted from Algerian myrtle leaves (0.33%; Djenane et al., 2011; Tuberoso, Barra, Angioni, Sarritzu, & Pirisi, 2006). Similarly to previous studies conducted on leaves and berries of Algerian and Italian *M. communis*, 1,8‐cineole was reported as one of the most important volatiles of *Mc*EO extracted from the Tunisian myrtle flowers (Djenane et al., 2011; Tuberoso et al., 2006). It seems obvious that the chemical variability in *Mc*EO of myrtle depends on the organ (leaves, berries, flower), the geographical origin, season of collection, and on edaphoclimatic conditions (Asllani, 2000; Ben Hsouna et al., 2017; Brada, Tabti, Boutoumi, Watheletc, & Lognayd, 2012; Chryssavgi, Vassiliki, Athanasios, Kibouris, & Michael, 2008; Pirbalouti, Mirbagheri, Hamedi, & Rahimi, 2014; Tuberoso et al., 2006). In fact, these factors affect the biosynthetic pathways of the myrtle, thus influencing the chemical composition of its *Mc*EOs, and their respective biological activities (Ben Hsouna et al., 2017; Brada et al., 2012).

**Table 1:** Chemical composition of *M. communis* flower essential oil (*Mc*EO)

No.	Components^a^	Rt (min)	KI	%^b^
1	α‐Pinene	9.18	939	35.20
2	β‐Pinene	10.45	980	0.24
3	Myrcene	10.80	991	1.21
4	Limonene	12.12	1,030	8.94
6	1,8‐cineole	12.26	1,033	17.00
7	Linalool	14.45	1,078	6.17
8	α‐Terpineol	17.64	1,090	3.86
9	Myrtenol	17.88	1,194	0.42
10	Linalyl acetate	19.73	1,257	0.85
11	Myrtenyl acetate	22.10	1,325	1.26
12	Terpenyl acetate	22.80	1,355	4.30
13	Geranyl acetate	23.80	1,385	4.42
14	Methyl eugenol	24.48	1,406	6.98
15	Transcaryophyllene	25.20	1,415	4.04
16	α‐Humulene	26.20	1,460	0.48
17	Carophyllene oxide	30.05	1,580	2.49
	Monoterpene hydrocarbons	46.07		
	Oxygenated monoterpenes	40.77		
	Sesquiterpenes	6.98		
	Total %	93.82		

Abbreviations: KI, Kovats Indices on HP‐5MS Capillary Column in reference to C_10_‐C_22_ *n*‐alkanes injected in the same conditions; Rt, retention time.

^a^ Identification of components based on GC‐MS Wiley 7.0 version library and National Institute of Standards and Technology 05 MS (NIST) library data.

^b^ %: Percentages are the means of two runs and were obtained from electronic integration measurements using a selective mass detector.

### 3.2 Cytotoxicity assay

**Figure 1:**
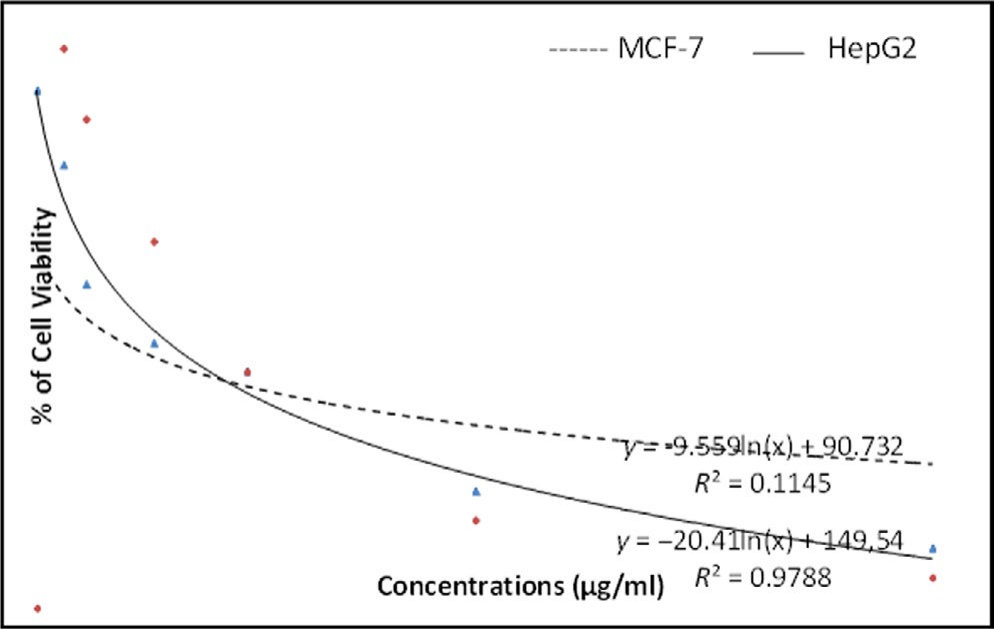
Cell viability of essential oil on MCF‐7 and HepG2 cell lines using MTT assay

*Mc*EO exhibited a significant concentration‐dependent cytotoxicity against HepG2 and MCF‐7 human cancer cell lines with IC_50_ values of 131.3 and 204.33 µg/ml, respectively (Figure 1). This is the first report on the cytotoxic activity of *Mc*EO against human cancer cell lines; nonetheless, the cytotoxic effect of essential oils extracted from different medicinal plants has been investigated (Innocenti et al., 2010). The essential oil major components such as limonene, terpinen‐4‐ol, and β‐Caryophyllene have been reported to exhibit an antitumor activity against different cell lines (Lu et al., 2014; Zhang, Scialis, Feng, & Leach, 2013). The cytotoxicity of essential oils could be attributed to various mechanisms including the disruption of the mevalonate pathway (Talib & Mahasneh, 2010), inducing of apoptosis (Kumar, D'Souza, Gaonkar, Rai, & Salimath, 2008) and the alteration of cell membranes, by either increasing its permeability and/or reducing the activity of its enzymes (Rezende, 2012; Zhang et al., 2013). While the cytotoxicity of *Mc*EO could be attributed to its major components (Lu et al., 2014; Zhang et al., 2013), minor components could contribute to that activity, individually or in synergy with major components.

### 3.3 Antioxidant activities

**Figure 2:**
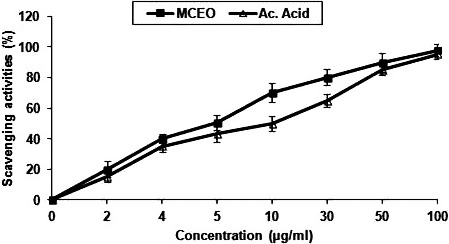
Scavenger effect of *Mc*EO at different concentrations, 0, 2, 4, 5, 10, 30, 50, and 100 µg/ml, on the stable 1,1‐diphenyl‐2‐picrylhydrazyl radical (DPPH). Results are expressed as percentage decrement of absorbance at 517 nm with respect to control. Ascorbic acid was used as a standard. Each value represents the mean ± *SD* (*n* = 3)

**Figure 3:**
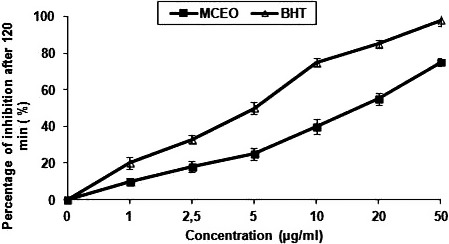
Antioxidant activities of *Mc*EO at different concentrations, 0, 1, 2.5, 5, 10, 20, and 50 µg/ml measured by β‐carotene bleaching method. BHT was used as standard. Values are means ± *SD* (*n* = 3)

The result of DPPH test (Figure 2) and those of β‐carotene bleaching assay (Figure 3) both showed that *Mc*EO had a strong dose‐dependent antioxidant activity. Notably, the antioxidant activity of *Mc*EO was more important (IC_50_ of 7.5 µg/ml) than that of ascorbic acid (IC_50_ = 8 µg/ml) in the DPPH scavenging assay. The antioxidant activity the *Mc*EO increased from 20% to 90.02% (DPPH radical scavenging activity assay compared to ascorbic acid as shown in Figure 2), and from 5% to 85% (β‐carotene bleaching assay compared to BHT as shown in Figure 3) when *Mc*EO concentrations increased from 2 to 50 µg/ml. The antioxidant activities of *Mc*EO may be mainly due to its major constituents and also to its unique chemical composition (Ben Hsouna, Hamdi, Ben Halima, & Abdelkafi, 2013; Dongmo et al., 2008; Ennajar et al., 2009; Guleria et al., 2012). Nonetheless, both minor and major components contribute to the biological activity of essential oils (Ben hsouna et al., 2013; Ennajar et al., 2009).

### 3.4 Antimicrobial activity

The antimicrobial activity of *Mc*EO was determined against Gram‐positive and Gram‐negative bacteria. Its potency was evaluated by test zone diameter of inhibition, MIC, MBC, and MBC/MIC values. According to our findings, test zone diameter of inhibition of *Mc*EO (50 µl/well) ranged between 14 and 22 mm and was comparable with that of gentamicin (10 μg/well), which ranged between 12 and 21 mm (Table 2). MIC and MBC results complied with the agar well diffusion results. Overall, MIC results showed that *Mc*EO induced an inhibition of all tested strains development; nonetheless, it was more efficient against Gram‐positive bacteria (MIC range: Gram‐positive 0.1%–0.4%; and Gram‐negative 0.78%–1.56%). Similarly, MBC values were also higher against the tested Gram‐negative strains (MBC range: Gram‐positive 0.78%–1.56%; and Gram‐negative 1.56%–3.12%; Table 2). The MBC/MIC ratio was determined to evaluate whether *McEO* were bactericidal or bacteriostatic. MBC/MIC ratios lower than four were considered indicative of a bactericidal activity, while MBC/MIC ratios higher than four were considered indicative of a bacteriostatic activity (Table 2; Soro et al., 2010). *Mc*EO exhibited a bacteriostatic activity against some Gram‐positive bacteria (Table 2). MBCs of those four strains required therefore higher multiples of MIC to show bactericidal effect. The recorded MBC in the remainder of the tested strains was similar to the MIC (indicating a good bactericidal activity against *B. cereus* ATCC 14579, *E. coli* ATCC 25922, *L. monocytogenes* ATCC 19117, *P. aeruginosa* ATCC 9027, and *S. enterica* ATCC 43972, with a ratio of MBC to MIC of 2 (Table 2).

**Table 2:** Zones of growth inhibition ± *SD* (mm), minimal inhibition concentration (MIC), and minimal bactericidal concentration (MBC) expressed in % (V/V) and ratio MBC/MIC showing antibacterial activity for *Mc*EO against human pathogenic bacteria compared to that of positive standard antibiotic (gentamicin)

Bacterial strains	Inhibition zones diameter ± *SD* (mm)^a^		MIC (%)(v/v)	MBC (%)(v/v)	MBC/MIC
	EO^b^	Gentamicin^c^			
Gram positive					
*B. subtilis* ATCC 6633	18 ± 0.7	20 ± 0.2	0.10 ± 0.7	0.78 ± 0.1	8
*B. cereus* ATCC 14579	22 ± 0.5	20 ± 0.4	0.39 ± 0.8	0.78 ± 0.3	2
*S. aureus* ATCC 25923	20 ± 0.7	25 ± 0.8	0.39 ± 0.4	1.56 ± 0.5	4
*S. epidermis* ATCC 12228	15 ± 0.4	20 ± 0.5	0.19 ± 0.4	1.56 ± 0.2	8
*E. faecalis* ATCC 29212	15 ± 0.5	12 ± 0.2	0.10 ± 0.7	0.78 ± 0.04	8
*L. monocytogenes* ATCC 19117	22 ± 0.4	15 ± 0.0	0.40 ± 0.2	0.8 ± 0.02	2
Gram negative					
*S.enterica* ATCC 43972	16 ± 0.6	18 ± 0.8	1.26 ± 0.3	3.12 ± 0.8	2
*E. coli* ATCC 25922	14 ± 0.3	21 ± 1.0	0.78 ± 04	1.56 ± 0.4	2
*P. aeruginosa* ATCC 9027	15 ± 0.5	18 ± 0.7	1.56 ± 0.5	3.12 ± 0.7	2

Note

Values are given as mean ± *SD* of triplicate experiments.

^a^ Diameter of inhibition zones of including diameter of disk 6 mm.

^b^ EO: *Myrtus communis* essential oil (50 µl/well).

^c^ The used concentration of gentamicin was 10 μg/well.

Similarly to the available literature on EOs (Ennajar et al., 2009; Prabuseenivasan, Jayakumar, & Ignacimuthu, 2006), the overall susceptibility data showed that Gram‐positive bacteria were more susceptible to *Mc*EO than Gram‐negative bacteria. Since the antimicrobial activity of essential oils has been attributed to their activity on the bacterial membrane (Cox et al., 2000), the recorded difference in susceptibility to *Mc*EO between Gram‐positive and Gram‐negative may be due to the difference in the cell envelope structure and composition (Shrivastava et al., 2007; Tamboli & Lee, 2013). It has been demonstrated that essential oils and their monoterpenoid components are responsible for that antimicrobial mechanism of action (Cox et al., 2000). Based on previous studies, the antimicrobial activity of *Mc*EO was attributed to its major components, including α‐Pinene (Stojkovic et al., 2008), 1.8‐Cineole (Akin, Demirci, Bagci, & Baser, 2010; Stojkovic et al., 2008), α‐Terpineol (Sun & Wu, 2007), and β‐Caryophyllene oxide (Ozturk & Ercisli, 2006). Similarly, the antimicrobial activity of *Mc*EO may be attributed to the high levels of α‐pinene (35.20%) and 1.8‐cineole (17%) present in its composition. Since α‐terpineol (3.86%) and β‐caryophyllene oxide (1.49%) were minor constituents of *Mc*EO, we assume that they act synergistically with major components to give the observed antimicrobial activity. Further studies are required to identify the *Mc*EO components responsible for its antimicrobial activity.

### 3.5 Microbiological characteristics

In treated samples, the mesophilic (APC), psychrotrophic (PTC) bacterial, and *Enterobacteriaceae* counts were lower than in controls (*p* < .05; Table 3). The APC counts of all six studied samples progressively augmented with the storage period. The initial APC value was of 2.40 log CFU/g, indicative of good meat quality. Based on the AFNOR V01‐003 (AFNOR, 2004) the end of microbiological shelf life of raw minced beef meat is limited by an APC count of 6.7 CFU/g. This value was surpassed by day 14 for T1, while the APC counts for T2, T3, T4, and T5 remained under the detection limits (log 6.7 CFU/g) until 21st day of storage.

**Table 3:** Effect of *Mc*EO and their combination with nisin on the microbial load of aerobic plate count (APC), psychrotrophic count (PTC), and *Enterobacteriaceae* count (log_10_ CFU/g) of raw minced meat beef during storage at 4°C

Days of storage at 4°C					
	0	3	7	14	21
APC					
C	2.40 ± 0.15	4.30 ± 0.35	5.66 ± 0.20	6.95 ± 0.31	7.58 ± 0.07
T_1_	2.40 ± 0.15	4.20 ± 0.28	5.60 ± 0.19	6.75 ± 0.20	6.90 ± 0.22
T_2_	2.40 ± 0.15	4.15 ± 0.06	5.02 ± 0.08	6.44 ± 0.13	6.10 ± 0.18
T_3_	2.40 ± 0.15	3.86 ± 0.19	4.70 ± 0.17	5.25 ± 0.26	5.60 ± 0.01
T_4_	2.40 ± 0.15	3.28 ± 0.20	3.83 ± 0.22	4.90 ± 0.15	5.66 ± 0.10
T_5_	2.40 ± 0.15	2.80 ± 0.17	3.00 ± 0.09	4.09 ± 0.12	5.25 ± 0.16
PTC count					
C	2.10 ± 0.12	4.86 ± 0.02	5.80 ± 0.05	7.20 ± 0.14	8.09 ± 0.19
T_1_	2.10 ± 0.12	4.38 ± 0.02	5.49 ± 0.01	6.20 ± 0.6	6.85 ± 0.12
T_2_	2.10 ± 0.12	4.37 ± 0.02	4.71 ± 0.02	5.33 ± 0.13	6.22 ± 0.24
T_3_	2.10 ± 0.12	4.00 ± 0.03	4.61 ± 0.01	5.10 ± 0.12	5.94 ± 0.18
T_4_	2.10 ± 0.12	2.66 ± 0.01	3.22 ± 0.17	4.20 ± 0.13	5.50 ± 0.19
T_5_	2.10 ± 0.12	2.61 ± 0.02	3.00 ± 0.02	3.40 ± 0.13	5.09 ± 0.21
*Enterobacteriaceae* count					
C	<1	2.30 ± 0.08	3.19 ± 0.01	3.80 ± 0.11	4.35 ± 0.08
T_1_	<1	2.00 ± 0.11	2.42 ± 0.14	3.60 ± 0.16	3.35 ± 0.18
T_2_	<1	1.70 ± 0.10	2.20v0.19	2.40 ± 0.11	3.20 ± 0.16
T_3_	<1	1.55 ± 0.11	1.88 ± 0.17	1.90 ± 0.15	2.20 ± 0.09
T_4_	<1	1.49 ± 0.11	1.62 ± 0.01	2.00 ± 0.11	2.09 ± 0.01
T_5_	<1	<1	<1	1.22 ± 0.12	1.66 ± 0.15

Note

Values are given as mean ± *SD* of triplicate experiments.

(T_0_): control/untreated sample, (T_1_): treatment with *Mc*EO at 0.4% v/w; (T_2_): treatment with *Mc*EO at 0.8% v/w; (T_3_): treatment with nisin at 500 AU/g; (T_4_): treatment with *Mc*EO at 0.4% v/w + nisin at 500 AU/g; (T_5_): treatment with *Mc*EO at 0.8% v/w + nisin at 500 AU/g.

PTC count of T2, T3, T4, and T5 was below the detectable levels of plate counts (log 6.7 CFU/g) after 21 days at 4°C (Table 3). Moreover, the treatment of samples with the combination *Mc*EO–nisin (T5: *Mc*EO at 0.8% v/w with nisin at 500 AU/g) was most effective in delaying the bacterial growth rate in meat. Similarly to Smaoui et al. (2016), the APC and PTC were lower in *Mc*EO‐treated meat than in controls. This observation may be attributed to the antioxidant activity of phenolic compounds present in essential oils (Joukar, Hosseini, Moosavi‐Nasab, Mesbahi, & Behzadnia, 2017; Ozogul et al., 2017). In fact, essential oils that exhibit an antimicrobial activity against foodborne pathogens are characterized with a high percentage of phenolic compounds (Joukar et al., 2017; Ozogul et al., 2017).

Following the addition of 0.4% (T1) and 0.8% (T2) *McEO*, a reduction in growth of *Enterobacteriaceae* was noted (in comparison with the control C; Table 3). Similarly, it has been shown that the addition of essential oils to meat was effective in reducing the *Enterobacteriaceae* count (Smaoui et al., 2016). Nonetheless, the combination of *McEO* and nisin is much more effective*.* The combination of *McEO* and nisin at 500 AU/g (T5) kept the level of *Enterobacteriaceae* below the detection limit which is 2 log10 CFU/g until the end of storage at 4°C (Table 3). The *Enterobacteriaceae* count was reduced to 1.66 log10 CFU/g after *McEO* addition at 0.8% combined with nisin at 500 AU/g (T5; Table 3). This is in accordance with the work of Smaoui et al. (2016) who reported that the combination of *Mentha piperita* essential oils and bacteriocin (named BacTN635) can be considered as an effective antimicrobial on minced beef meat during refrigerated storage (Smaoui et al., 2016).

### 3.6 Kill‐time analysis: effect of *Mc*EO (alone and in combination with nisin)

**Figure 4:**
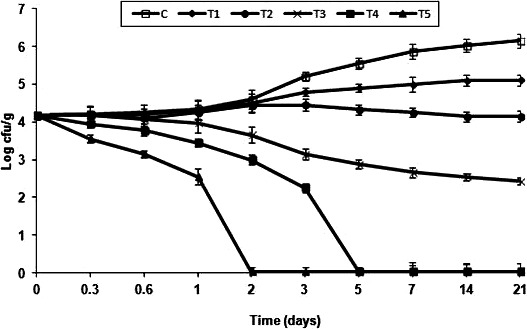
Effect of *Mc*EOs and their combination with nisin on the *Listeria monocytogenes* ATCC 19117 (log10 CFU/g) of raw minced meat beef during storage at 4°C. (C): control, (T1): treatment with *Mc*EO at 0.4% v/w; (T2): treatment with *Mc*EO at 0.8% v/w; (T3): treatment with nisin at 500 AU/g; (T4): treatment with *Mc*EO at 0.4% v/w + nisin at 500 AU/g; (T5): treatment with *Mc*EO at 0.8% v/w + nisin at 500 AU/g. Values are means ± *SD* (*n* = 3)

The impact of *Mc*EOs tested individually and in mixture with nisin) on the growth of *L. monocytogenes* ATCC 19117 in raw minced meat beef stored at 4°C is shown in Figure 4. The results of viable count of *L. monocytogenes* in meat after treatment with *Mc*EOs were in agreement with prior in vitro results (Section 3.4).

All the results are reported during raw minced meat beef storage at 4°C. At day 0, the number of *L. monocytogenes* was comparable for all samples (*p* > .05). The number of *L. monocytogenes* in control was higher than for all *Mc*EO‐treated samples (alone and in combination with nisin) over all the storage period. After 21 days of storage, a reduction by 2.0 log cycles of *L. monocytogenes* count was recorded in samples treated with 500 AU/g nisin (T3). Moreover, the use of *Mc*EO alone at 0.4% and 0.8% (T1 and T2) delayed *L. monocytogenes* growth (*p* < .05) during storage. The mixture *Mc*EOs/nisin reduced the growth of *L. monocytogenes* remarkably after 7 days. That combination also reduced *L. monocytogenes* count by 7.25 log units below the control after 2 days and stabilized the concentration of *L. monocytogenes* for 21 days (Figure 4).

### 3.7 Physicochemical characteristics

We noted alterations in the pH values of raw minced beef meat samples (C, T_1‐5_) during storage period at 4°C (Table 4). The initial pH of minced beef meat samples (C, T_1‐5_) was of 5.60. The pH value of the control sample (C) increased the most among all the samples (increase in pH from 5.60 ± 0.26 to 6.92 ± 0.24, with a difference of about 1.37 in pH between day 21 and day 0 of storage). The pH increase in treated minced beef samples (T_1‐5_) was lower than that of the control sample (C). The lowest pH values increase over the period of storage (difference in recorded pH values between day 21 and day 0) was recorded for T_5_ (about 0.51 in pH difference, which was < T_4_<T_3_ < T_2_ < T_1_ < C). This indicates that *McEO*‐treated samples were preserved better than untreated ones, with the best preservation conditions seen in samples treated with *Mc*EO at 0.8% combined with nisin at 500 AU/g (T_5_). The antimicrobial activity of *Mc*EO (Table 3) reduced the development of lactic acid bacteria causing a delay in meat spoilage (Sharma et al., 2017; Smaoui et al., 2016), and subsequent pH changes.

**Table 4:** Effect of McEO and their combination with nisin on pH and TBARS (in mg of malonaldehyde equivalents per kg of sample [mg of MDA/kg]) values of raw minced meat beef during storage at 4°C

Days of storage at 4°C					
	0	3	7	14	21
pH					
C	5.61 ± 0.26	5.76 ± 0.22	6.32 ± 0.17	6.80 ± 0.24	6.98 ± 0.24
T_1_	5.60 ± 0.27	5.70 ± 0.21	6.29 ± 0.19	6.30 ± 0.20	6.62 ± 0.25
T_2_	5.60 ± 0.21	5.66 ± 0.20	6.24 ± 0.20	6.30 ± 0.19	6.61 ± 0.20
T_3_	5.58 ± 0.21	5.62 ± 0.22	6.00 ± 0.17	6.11 ± 0.14	6.41 ± 0.25
T_4_	5.58 ± 0.20	5.54 ± 0.15	5.90 ± 0.13	6.00 ± 0.16	6.21 ± 0.22
T_5_	5.58 ± 0.19	5.58 ± 0.11	5.75 ± 0.21	5.88 ± 0.23	6.09 ± 0.14
TBARS (in mg of MDA/kg)					
C	0.20 ± 0.10	1.66 ± 0.11	2.00 ± 0.19	2.75 ± 0.20	3.95 ± 0.15
T_1_	0.20 ± 0.10	1.35 ± 0.10	1.90 ± 0.20	2.09 ± 0.22	2.30 ± 0.11
T_2_	0.20 ± 0.10	1.35 ± 0.1	1.90 ± 0.16	1.94 ± 0.16	2.30 ± 0.13
T_3_	0.20 ± 0.10	0.70 ± 0.09	1.15 ± 0.12	1.70 ± 0.20	2.30 ± 0.09
T_4_	0.20 ± 0.10	0.64 ± 0.11	1.13 ± 0.19	1.45 ± 0.14	2.28 ± 0.11
T_5_	0.20 ± 0.10	0.54 ± 0.10	0.89 ± 0.08	1.15 ± 0.10	1.80 ± 0.12

Note

Values are given as mean ± *SD* of triplicate experiments

(C): control; (T_1_): treatment with *Mc*EO at 0.4% v/w; (T_2_): treatment with *Mc*EO at 0.8% v/w; (T_3_): treatment with nisin at 500 IU/g; (T_4_): treatment with *Mc*EO at 0.4% v/w + nisin at 500 AU/g; (T_5_): treatment with *Mc*EO at 0.8% v/w + nisin at 500 IU/g.

Similar observation was made for the oxidation of lipids in minced beef meat samples (C, T_1‐5_). Initial TBARS values recorded for all samples were the same for all the samples (~0.20 MDA/kg). Moreover, TBARS increased the most in the control sample C (from 0.20 ± 0.10 to 3.95 ± 0.15 mg MDA/kg, with a difference of about 3.75 mg of MDA/kg in TBARS value between day 21 and day 0) and the least in T_5_ sample (from 0.20 ± 0.10 to 1.80 ± 0.12 mg MDA/kg, with a difference of about 1.6 mg of MDA/kg in TBARS value between day 21 and day 0) over the storage period (day 0–21; Table 4). The threshold for the acceptability of TBARS is 2 (Campo et al., 2006). The TBARS recorded for T_5_ was <2, indicative of the protective antioxidant effect of the combination used in T_5_. The rapid rate of increase in lipid oxidation in the control sample (C) can be attributed to the absence of antioxidants (Ben Hsouna et al., 2017). The antioxidant activity of *Mc*EO (alone or in combination with nisin) slowed down the free radical propagation process and the oxidation rate (TBARS < 2.3 in T_1‐5_; Table 4).

### 3.8 Sensory scores

Table 5 summarized the findings relative to the sensory analysis of minced beef meat treated with essential oil tested individually or in mixture. Sensory evaluation showed a decrease in all groups evaluated sensory attributes (color, odor, and appearance and overall acceptability) during the storage. These results revealed a best sustainability for the group treated with the combination *Mc*EO at 0.8% v/w + nisin at 500 IU/g (T_5_) followed by T_4_ (*Mc*EO at 0.4% v/w with nisin at 500 AU/g). For the samples treated with *Mc*EO and *Mc*EO/nisin combinations, color scores above five corresponding to the rejection limit of raw minced beef meat were not reached until the 14th day of storage. The overall acceptability of treated minced beef (T_1‐5_) was maintained for 14 days (*p* < .05). The untreated control sample (C) was unacceptable from day 7 (*p* < .05). The decrease in overall acceptability scores during storage (C, T_1‐5_) might be due to a decline in scores of other sensory attributes or characteristics such as color, odor, and appearance (Smaoui et al., 2016).

**Table 5:** Effect of *Mc*EO and their combination with nisin on color, odor, and overall acceptability of raw minced meat beef stored at 4°C

Days of storage at 4°C					
	0	3	7	14	21
Color					
C	6.02 ± 0.29	5.80 ± 0.11	4.50 ± 0.15	3.15 ± 0.09	1.20 ± 0.17
T_1_	6.45 ± 0.22	6.30 ± 0.15	6.00 ± 0.23	5.00 ± 0.14	3.55 ± 0.09
T_2_	6.45 ± 0.22	6.30 ± 0.12	6.15 ± 0.23	5.00 ± 0.21	3.44 ± 0.11
T_3_	6.50 ± 0.16	6.45 ± 0.17	6.25 ± 0.21	5.25 ± 0.21	3.50 ± 0.15
T_4_	6.80 ± 0.17	6.45 ± 0.21	6.33 ± 0.29	5.33 ± 0.22	3.50 ± 0.09
T_5_	6.90 ± 0.20	6.85 ± 0.22	6.66 ± 0.26	5.80 ± 0.24	3.80 ± 0.12
Odor					
C	5.07 ± 0.18	5.15 ± 0.13	4.35 ± 0.11	2.33 ± 0.10	1.00 ± 0.01
T_1_	5.07 ± 0.15	5.15 ± 0.15	5.00 ± 0.13	5.44 ± 0.19	2.33 ± 0.08
T_2_	5.07 ± 0.16	5.15 ± 0.12	5.00 ± 0.13	5.44 ± 0.21	3.11 ± 0.07
T_3_	5.58 ± 0.14	5.22 ± 0.17	5.55 ± 0.21	5.44 ± 0.19	2.33 ± 0.06
T_4_	5.77 ± 0.11	5.67 ± 0.21	5.55 ± 0.19	5.55 ± 0.22	3.11 ± 0.04
T_5_	6.50 ± 0.19	6.00 ± 0.22	5.55 ± 0.18	5.55 ± 0.23	3.11 ± 0.02
Overall acceptability					
C	6.10 ± 0.12	5.70 ± 0.16	5.00 ± 0.16	2.85 ± 0.16	1.10 ± 0.01
T_1_	6.50 ± 0.19	6.25 ± 0.25	5.33 ± 0.23	5.00 ± 0.16	3.10 ± 0.08
T_2_	6.50 ± 0.23	6.33 ± 0.23	5.95 ± 0.22	5.55 ± 0.20	3.15 ± 0.12
T_3_	6.50 ± 0.24	6.45 ± 0.20	6.00 ± 0.25	5.50 ± 0.26	3.15 ± 0.16
T_4_	6.50 ± 0.23	6.50 ± 0.20	6.50 ± 0.19	5.80 ± 0.2	3.50 ± 0.15
T_5_	6.85 ± 0.24	6.80 ± 0.20	6.65 ± 0.20	5.80 ± 0.23	3.70 ± 0.15

Note

Values are given as mean ± *SD* of triplicate

(C): control, (T_1_): treatment with *Mc*EO at 0.4% v/w; (T_2_): treatment with *Mc*EO at 0.8% v/w; (T_3_): treatment with nisin at 500 AU/g; (T_4_): treatment with *Mc*EO at 0.4% v/w + nisin at 500 AU/g; (T_5_): treatment with *Mc*EO at 0.8% v/w + nisin at 500 AU/g.

## 4 CONCLUSION

In summary, *Mc*EO inhibited the growth of all the bacterial strains tested at noncytotoxic concentrations. *Mc*EO exhibited a more potent antimicrobial activity in the case of Gram‐positive bacteria. The effect of the *Mc*EO against *L. monocytogenes* was bactericidal. Moreover, *Mc*EO also exhibited a potent antioxidant activity. Based on its antimicrobial and antioxidant activities, *Mc*EO can serve as a natural preservative for minced beef meat.

*Mc*EO (at 0.4% and 0.8%) exhibited antilisterial and antioxidant activities. Its combination with nisin enhanced the potency of the aforementioned biological effects. Among the different treatments tested (T_1‐5_), “0.8% *Mc*EO /500 IU/g of nisin” was the most effective in inhibiting the growth of all the tested strains, including that of *L. Monocytogenes*. Our results indicate that the combination of 0.8% *Mc*EO with 500 IU/g nisin was the most efficient in preventing lipid oxidation, extending the shelf life, and improving sensory quality attributes of minced beef meat during refrigeration (4°C). Further investigation of the combination of *Mc*EO with nisin for the development of natural food preservatives is needed.

## CONFLICT OF INTEREST

All authors declare that there is no conflict of interest.

## ETHICAL STATEMENT

There was no human or animal testing in this study.
